# When Bipolar Disorder Meets Obsessive-Compulsive Disorder: A Perfect Storm for Cognitive and Functional Impairment

**DOI:** 10.1192/j.eurpsy.2026.11097

**Published:** 2026-07-31

**Authors:** E. M. Bahri, S. Ellini, F. Ghrissi, W. Cherif, R. Damak, M. E. Cheour, F. F. Romdhane

**Affiliations:** 1 Espoir Addiction Service, Jbel Ouest Healthcare Complex, Zaghouan; 2 Psychiatry Department E, Razi Hospital, Manouba, Tunisia

## Abstract

**Introduction:**

Comorbid anxiety disorders are highly prevalent in Bipolar Disorder (BD) and are associated with a more severe illness course. However, the specific impact of Obsessive-Compulsive Disorder (OCD) on cognitive and functional outcomes in BD remains underexplored. It is critical to understand if this particular comorbidity confers an additional burden, guiding more precise treatment strategies.

**Objectives:**

This study investigates the relationship between comorbid OCD and the levels of subjective cognitive complaints and functional impairment in patients with euthymic BD Type I.

**Methods:**

In a sample of 100 euthymic patients with BD Type I, we assessed psychiatric comorbidities through clinical interview. Participants completed the Cognitive Complaints in Bipolar Disorder Rating Assessment (COBRA) scale and the Functioning Assessment Short Test (FAST). Patients with comorbid OCD (n=9) were compared to those without (n=91) on their COBRA and FAST scores using non-parametric Mann-Whitney U tests. The analysis was controlled for other anxiety comorbidities, which showed no significant effects. Data was collected and analyzed using SPSS 27 software.

**Results:**

The patient sample (n=100) was evenly balanced, comprising 53 women (53%) and 47 men (47%). The mean age was 42.5 years (±10.8). A majority of patients (n=60, 60%) were unemployed, highlighting the significant functional impact of BD. Over half of the patients (n=53, 53%) were single. Most patients (n=76, 76%) had a secondary level of education or lower. The median duration of illness was 18 years [IQR 9-26]. A high proportion of patients (n=85, 85%) had a history of psychotic symptoms, underscoring the severity of the cohort. Half of the patients (n=50, 50%) reported a history of at least one suicide attempt. Rapid Cycling was present in a minority of patients (n=10, 10%).

Within this cohort, the subset of patients with comorbid OCD (n=9) reported significantly higher subjective cognitive complaints than those without (Median COBRA score: 16 vs. 8; p=0.038). Comorbid OCD was also associated with significantly greater global functional impairment (Mean FAST score: 39.7 vs. 27.5; p=0.021). Other anxiety disorder comorbidities did not show statistically significant associations with either COBRA or FAST scores.

**Conclusions:**

Comorbid OCD in BD type 1 is associated with a significantly greater burden of both subjective cognitive complaints and functional impairment, even during euthymic periods. This suggests that OCD comorbidity may identify a distinct and more severe subpopulation within the BD spectrum. The findings highlight the necessity for integrated treatment approaches that simultaneously address BD mood stability, OCD symptoms, and functional remediation. Clinicians should actively screen for OCD in BD patients and consider its presence a marker for needing intensified cognitive and functional support.

**Disclosure of Interest:**

None Declared

